# Dopaminergic Treatment Effects on Dysarthric Speech: Acoustic Analysis in a Cohort of Patients With Advanced Parkinson's Disease

**DOI:** 10.3389/fneur.2020.616062

**Published:** 2021-02-05

**Authors:** Francesco Cavallieri, Carla Budriesi, Annalisa Gessani, Sara Contardi, Valentina Fioravanti, Elisa Menozzi, Serge Pinto, Elena Moro, Franco Valzania, Francesca Antonelli

**Affiliations:** ^1^Neurology Unit, Neuromotor and Rehabilitation Department, Azienda USL - IRCCS di Reggio Emilia, Reggio Emilia, Italy; ^2^Clinical and Experimental Medicine PhD Program, University of Modena and Reggio Emilia, Modena, Italy; ^3^Department of Biomedical, Metabolic and Neural Sciences, University of Modena and Reggio Emilia, Modena, Italy; ^4^Azienda Ospedaliero Universitaria di Modena, Modena, Italy; ^5^Department of Clinical and Movement Neurosciences, UCL Queen Square Institute of Neurology, London, United Kingdom; ^6^Aix Marseille Univ, CNRS, LPL, Aix-en-Provence, France; ^7^Division of Neurology, Centre Hospitalier Universitaire (CHU), Grenoble Alpes University, Grenoble Institute of Neurosciences, Grenoble, France

**Keywords:** acoustic analysis, dysarthria, dyskinesias, levodopa, Parkinson's disease, speech

## Abstract

**Importance:** The effects of dopaminergic treatment on speech in patients with Parkinson's disease (PD) are often mixed and unclear. The aim of this study was to better elucidate those discrepancies.

**Methods:** Full retrospective data from advanced PD patients before and after an acute levodopa challenge were collected. Acoustic analysis of spontaneous monologue and sustained phonation including several quantitative parameters [i.e., maximum phonation time (MPT); shimmer local dB] as well as the Unified Parkinson's Disease Rating Scale (UPDRS) (total scores, subscores, and items) and the Clinical Dyskinesia Rating Scale (CDRS) were performed in both the defined-OFF and -ON conditions. The primary outcome was the changes of speech parameters after levodopa intake. Secondary outcomes included the analysis of possible correlations of motor features and levodopa-induced dyskinesia (LID) with acoustic speech parameters. Statistical analysis included paired *t*-test between the ON and OFF data (calculated separately for male and female subgroups) and Pearson correlation between speech and motor data.

**Results:** In 50 PD patients (male: 32; female: 18), levodopa significantly increased the MPT of sustained phonation in female patients (*p* < 0.01). In the OFF-state, the UPDRS part-III speech item negatively correlated with MPT (*p* = 0.02), whereas in the ON-state, it correlated positively with the shimmer local dB (*p* = 0.01), an expression of poorer voice quality. The total CDRS score and axial subscores strongly correlated with the ON-state shimmer local dB (*p* = 0.01 and *p* < 0.01, respectively).

**Conclusions:** Our findings emphasize that levodopa has a poor effect on speech acoustic parameters. The intensity and location of LID negatively influenced speech quality.

## Introduction

Hypokinetic dysarthria is a very common motor feature in Parkinson's disease (PD) being reported in 70–90% of patients (1). It is characterized by monopitch and monoloudness, breathy or harsh voice, reduced loudness, imprecise articulation, abnormalities of pause ratio and speech rate, and airflow insufficiency (2, 3). Hypokinetic dysarthria can occur at any stage of PD and becomes worse as the disease progresses, with loss of functional communication and a significant negative impact on patients' quality of life (4). Mixed results have been reported regarding the effects of dopaminergic treatment on speech in PD patients (5–7). After levodopa intake, some improvement has been found on vowel articulation (only in individual patients), increased loudness, faster rate (with a variable effect on the final acoustic product of speech), global voice quality, and stop consonant articulation (7–12). Two recent meta-analyses concluded that levodopa therapy modifies only mean fundamental frequency, jitter, and shimmer with little or no effects on vocal intensity in PD patients (6, 7). Other studies found no improvement in phonation, intonation, articulation, and speech velocity parameters after levodopa intake, neither under short-term levodopa administration nor on stable dopaminergic treatment (8, 13). Moreover, chronic levodopa administration has been associated with more dysfluent events after 3–6 years of treatment (14). This inconsistency in results may be caused (at least partially) by different pathophysiological mechanisms underlying appendicular and axial PD motor symptoms (5). Indeed, axial symptoms, such as dysarthria, swallowing troubles, gait disorders, and postural instability, are considered resulting from non-dopaminergic (i.e., cholinergic) lesions affecting brain areas (15). On the other hand, limb symptoms (i.e., tremor, rigidity, or bradykinesia) are related principally to dopaminergic lesion and are consequently levodopa-responsive symptoms (15). Knowledge about the influence of levodopa-induced dyskinesia (LID) on speech, and the possible relationship between such involuntary movements and specific acoustic parameters, is still incomplete. To fill this gap, the objective of this study was to assess possible correlations between PD motor features, LID, and acoustic speech parameters in a large cohort of advanced PD patients before and after an acute levodopa challenge.

## Methods

### Participants

We retrospectively analyzed data from a cohort of consecutive advanced PD patients admitted at the Neurology Department of the Sant'Agostino Estense Hospital, Modena, Italy, from 2012 to 2017 for a preoperative evaluation before subthalamic nucleus deep brain stimulation (STN-DBS) surgery. All patients fulfilled the criteria of idiopathic PD according to the UK Brain Bank criteria (16) and complained about disabling motor complications (i.e., motor fluctuations or levodopa-induced dyskinesia) at time of evaluation. Non-native Italian speakers and patients with severe cognitive impairment were excluded from the analysis. The study was approved by the local ethics committee (protocol number: 0031287/18), and written informed consent was obtained from participants according to the Declaration of Helsinki (17).

### Clinical Assessment

The clinical evaluation was performed in accordance with the Core Assessment Program for Surgical Interventional Therapies in Parkinson's Disease (CAPSIT-PD) protocol (18). Each patient underwent an acute levodopa challenge to evaluate levodopa responsiveness. Disease severity was assessed using the Hoen and Yahr scale (H&Y) and the four parts of the Unified Parkinson's Disease Rating Scale (UPDRS) (19) in the defined “OFF” condition (obtained after a 12-h antiparkinsonian medication withdrawal) and in the defined “ON” condition (obtained after 60 min and the administration of a 30% higher dose of the usual levodopa morning intake) (18). The ON-state LID was assessed applying the Clinical Dyskinesia Rating Scale (CDRS) (20).

### Speech Evaluation

The patients' speech was evaluated during the levodopa challenge (in the OFF and ON conditions) by two speech and language therapists (CB and AG) with expertise in phonetics and movement disorder-related speech disturbances. Furthermore, speech data were always retrospectively analyzed by the same two evaluators (CB and AG) through the years. Speech assessment in the ON condition was always performed ~1 h after the levodopa intake, immediately at the end of neurological examination, thus allowing to test patients in their best ON condition. Evaluations were made in a quiet room. Speech was recorded using a digital voice recorder (model SONY ICDPX240), maintained at 20 cm from the patients' lips. Speech tasks included a 30-s spontaneous monologue and the sustained phonation of a vowel (patients were asked to produce the phoneme */a/* for as long as they could). The total speech evaluation time was 15 min for each condition. Acoustic analysis was performed using the open-source Praat software® (21). The following parameters were calculated: mean intensity of spontaneous speech expressed in decibel (dB); mean fundamental frequency (F0) and mean standard deviation of F0 (F0 SD) of spontaneous speech, in hertz (Hz); maximum phonation time (MPT), in seconds; mean intensity of sustained phonation, in dB; shimmer of sustained phonation (in dB); mean F0 of sustained phonation (in Hz); jitter of sustained phonation (in Hz); mean noise-to-harmonics ratio (NHR) of sustained phonation (in dB); and fraction of locally unvoiced frames of sustained phonation (in %). We selected these parameters because they represent acoustic characteristics previously reported as altered in hypokinetic dysarthria (8, 22). In particular, alterations of such acoustic characteristics are expected to be related to different voice dysfunctions: reduced mean intensity of both spontaneous speech and sustained phonation would correlate with reduced loudness; mean F0 and F0 SD of spontaneous speech with monopitch; shorter maximum phonation time with airflow insufficiency; and changes in the jitter (frequency instability of the vocal folds), shimmer (amplitude instability of the vocal folds), and NHR would be associated with harsh voice and poorer voice quality (8, 22).

### Statistical Analysis

The primary outcome of the study was the changes of all the different speech parameters in the OFF- and ON-medication conditions. Secondary outcomes included the analysis of a possible correlation of motor features and LID with acoustic speech parameters. The following UPDRS subscores were calculated, as sums of the determined items, both in the defined-OFF and defined-ON conditions: speech subscore (UPDRS-II, item 5; UPDRS-III, item 18) and postural instability/gait disorders (PIGD) subscore (UPDRS-II, items 13–15; UPDRS-III, items 29–30). Moreover, the dyskinesia subscore (UPDRS-IV, items 32–34) was calculated. Item 18 of the UPDRS-III motor speech was also separately included in the analysis. Concerning the LID, we calculated both the total CDRS score (range 0–28) and the axial CDRS dyskinesia subscore (face including tongue, neck, and trunk; range 0–12) in the ON condition. The total amount of chronic antiparkinsonian medications was calculated as levodopa equivalent daily dose (LEDD) milligrams (mg) according to previously reported conversion factors (23). Because the variables were widely normally distributed (Kolmogorov–Smirnov test), *T* test for paired samples was applied to find significant changes after levodopa intake in motor scores (calculated in the entire cohort) and in speech parameters (calculated separately for both male and female subgroups). Furthermore, the Pearson correlation test was applied to evaluate correlations between the UPDRS part III total score and subscores (speech item, speech subscore, and PIGD subscore), CDRS score and axial CDRS subscore, and speech parameters both in the OFF and ON conditions. Continuous variables were expressed as mean (±SD) and median (range), while frequencies and percentage were calculated for categorical variables. A *p*-value <0.05 was considered significant. Statistical analysis was performed using the IBM SPSS Statistics for Windows version 20.0 (IBM, Armonk, NY, USA).

## Results

### Demographic and Clinical Results

From a total of 63 consecutive advanced PD patients, we excluded from the analyses 13 patients for the following reasons: non-native Italian speakers (five patients), missing data (four patients), and severe cognitive impairment (four patients). Demographic and clinical characteristics of the remaining 50 patients are shown in Table 1. As expected, levodopa improved significantly the motor performances of our patients (Table 2), with a significant reduction of the UPDRS part-III total score (*p* < 0.001), the PIGD subscore (*p* < 0.001), the speech subscore (*p* < 0.001), and the UPDRS III item 18 (speech item) (*p* < 0.001).

**Table 1:** Patients' demographic and clinical characteristics.

**Variable**	**Total *n* = 50** ***n* (%); mean (±SD)**
Age	60.62 (±6.97)
**Sex**	
◦Male	32 (64)
◦Female	18 (36)
Age at PD onset (years)	49.78 (±6.97)
Disease duration (years)	10.84 (±4.44)
UPDRS part I	2.5 (±2.03)
UPDRS part IV	6.82 (±2.49)
UPDRS dyskinesia subscore	2.52 (±1.75)
LEDD (milligrams)	1092.94 (±446.55)
Total CDRS score (0–28)	6.34 (±4.62)
Axial CDRS subscore (0–12)	2.38 (±2.19)

*CDRS, Clinical Dyskinesia Rating Scale; LEDD, levodopa equivalent daily dose; UPDRS, Unified Parkinson's Disease Rating Scale; PD, Parkinson's disease*.

**Table 2:** Changes of clinical variables after levodopa intake.

**Variable**	**OFF**	**ON**	**Mean change after levodopa intake (95% CI)**	***p*-Value**
	**Total *n* = 50**	**Total *n* = 50**		
	**Mean (±SD)**	**Mean (±SD)**		
UPDRS part II	21.59 (±7.43)	8.63 (±4.96)	12.95 (11.04–14.87)	<0.001
UPDRS part III	40.00 (±13.50)	15.78 (±7.57)	24.22 (21.27–27.16)	<0.001
UPDRS III item 18 (speech item)	1.68 (±0.68)	0.94 (±0.71)	0.74 (0.58–0.90)	<0.001
PIGD subscore	9.48 (±4.14)	3.78 (±2.89)	5.70 (4.72–6.67)	<0.001
Speech subscore	3.32 (±1.31)	1.72 (±1.32)	1.60 (1.24–1.95)	<0.001

*UPDRS, Unified Parkinson's Disease Rating Scale; PIGD, Postural Instability Gait Disorders*.

### Primary Outcome

#### Changes of Speech Parameters During Levodopa Challenge

There was a significant effect only in the MPT of sustained phonation in the female subgroup, which was significantly increased by levodopa intake [mean increment: 2.72 s; 95% confidence interval (CI) 1.17–4.27; *p* = 0.002]. No statistical differences were seen in all other acoustic measurements between the OFF- and ON-medication conditions (Table 3).

**Table 3:** Changes of speech acoustic variables after levodopa intake in male and female patients.

**Variable**		**OFF**	**ON**	**Mean change after levodopa intake** **(95% CI)**	***p*-Value**
		**Mean (±SD)**	**Mean (±SD)**		
Mean intensity of spontaneous speech (dB)	M (*n* = 32)	65.81 (±5.85)	65.09 (±8.05)	0.71 (−1.29 to 2.72)	0.47
	F (*n* = 18)	64.89 (±6.45)	64.61 (±7.89)	0.27 (−1.19 to 1.75)	0.69
Mean F0 of spontaneous speech (Hz)	M (*n* = 32)	127.78 (±23.80)	130.07 (±26.62)	−2.29 (−7.96 to 3.37)	0.41
	F (*n* = 18)	185.77 (±18.77)	180.50 (±24.36)	5.27 (−5.90 to 16.45)	0.33
F0 SD of spontaneous speech (Hz)	M (*n* = 32)	32.58 (±21.39)	36.63 (±24.20)	−4.04 (−10.62 to 2.52)	0.21
	F (*n* = 18)	34.07 (±16.68)	33.50 (±12.18)	0.56 (−8.86 to 10.00)	0.90
MPT of sustained phonation (seconds)	M (*n* = 32)	13.63 (±5.23)	15.50 (±5.59)	−1.74 (−3.60 to 0.11)	0.06
	F (*n* = 18)	9.00 (±3.67)	11.72 (±4.59)	−2.72 (−4.27 to −1.17)	**0.002**
Mean intensity of sustained phonation (dB)	M (*n* = 30)	73.45 (±6.01)	71.43 (±7.25)	1.83 (−0.09 to 3.76)	0.06
	F (*n* = 17)	70.24 (±8.26)	70.47 (±6.81)	–.23 (−3.16 to 2.69)	0.86
Mean frequency of sustained phonation (Hz)	M (*n* = 28)	135.90 (±34.80)	130.43 (±38.62)	3.48 (−8.11 to 15.07)	0.54
	F (*n* = 15)	193.47 (±41.85)	194.69 (±22.60)	0.40 (−15.73 to 16.53)	0.95
Jitter local of sustained phonation (%)	M (*n* = 28)	0.76 (±0.70)	0.77 (±0.57)	0.01 (−0.29 to 0.32)	0.91
	F (*n* = 16)	0.87 (±0.51)	0.092 (±0.67)	−0.08 (−0.50 to 0.32)	0.65
Shimmer local of sustained phonation (dB)	M (*n* = 28)	0.83 (±0.30)	0.87 (±0.32)	−0.02 (−0.14 to 0.10)	0.72
	F (*n* = 16)	0.79 (±0.39)	0.83 (±0.33)	−0.05 (−0.27 to 0.15)	0.56
Mean NHR of sustained phonation (dB)	M (*n* = 28)	0.13 (±0.12)	0.18 (±0.23)	−0.03 (−0.12 to 0.04)	0.36
	F (*n* = 16)	0.12 (±.08)	0.16 (±.16)	−0.04 (−0.16 to 0.06)	0.38
Fraction of locally unvoiced frames of sustained phonation	M (*n* = 28)	3.03 (±8.15)	1.78 (±4.03)	1.39 (−1.02 to 3.81)	0.24
	F (*n* = 16)	3.50 (±6.53)	3.80 (±6.25)	−0.40 (−5.72 to 4.91)	0.87

*dB, decibel; F0, fundamental frequency; Hz, hertz; MPT, maximum phonation time; NHR, noise-to-harmonics ratio; F0 SD, standard deviation of fundamental frequency. The bold means that this p-value (0.002) has a statistical significance*.

### Secondary Outcomes

#### Correlation of Motor Features With Speech Parameters

In the OFF-medication condition, the UPDRS III speech item correlated negatively with the MPT (*p* = 0.02, *r*-value: −0.30; 95% CI −0.54 to −0.03), meaning that patients with a high speech score in the OFF condition had an increased airflow insufficiency (reduced MPT) in the same condition. In the ON-medication condition, the UPDRS III speech item correlated positively with the shimmer ON medication (*p* = 0.01, *r*-value: 0.37; 95% CI 0.08–0.60), meaning that patients with higher speech score after levodopa intake showed a higher amplitude instability of the vocal folds, indicative of a poorer voice quality. Furthermore, the ON-medication speech score correlated negatively with the OFF-medication MPT (*p* < 0.01, *r*-value: −0.37; 95% CI −0.59 to −0.10), meaning that patients with higher speech score in the ON condition presented a reduced MPT before levodopa intake. The UPDRS axial subscore in the ON medication correlated positively with higher mean F0 values of sustained phonation (*p* < 0.01, *r*-value: 0.41; 95% CI 0.13–0.63) and mean F0 values of spontaneous speech (*p* < 0.01, *r*-value: 0.40; 95% CI 0.01–0.05), meaning that patients with higher axial impairment after levodopa intake showed higher mean frequency of both sustained phonation and spontaneous speech in the ON-medication condition. In the OFF condition, the UPDRS III speech item negatively correlated with the intensity of sustained phonation in the ON condition (*p* = 0.03, *r*-value: −0.31; 95% CI −0.55 to −0.02), meaning that patients with a high speech score in the OFF condition had a reduction of voice loudness (reduced intensity of sustained phonation) after medication intake.

#### Correlation of Speech Parameters With LID

**Figure 1:**
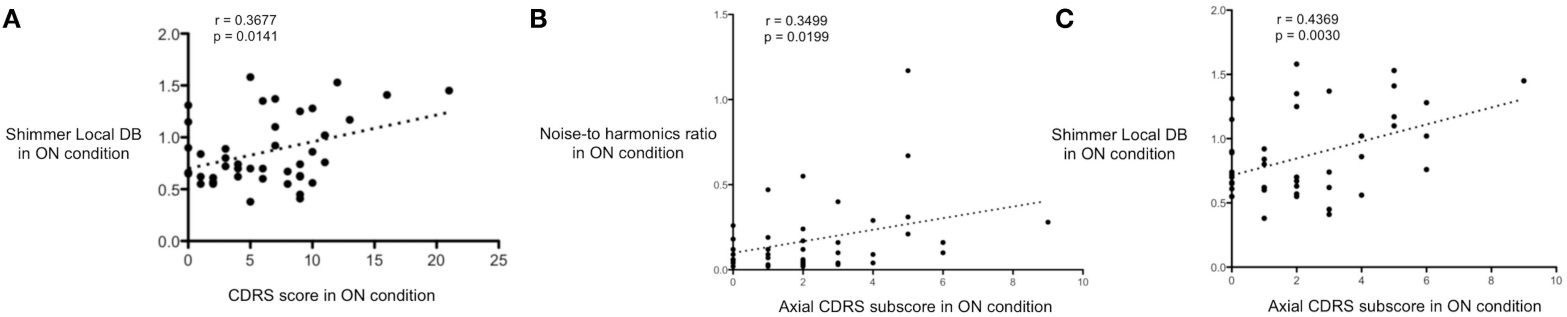
Correlation between intensity and location of LID and acoustic speech parameters. **(A)** Significant positive correlation between Clinical Dyskinesia Rating Scale (CDRS) total score (x-axis) and shimmer local DB (y-axis) in the ON condition. **(B)** Significant positive correlation between axial CDRS subscore (x-axis) and noise-to-harmonics ratio (y-axis) in the ON condition. **(C)** Statistically positive correlation between axial CDRS subscore (x-axis) and shimmer local DB (y-axis) in the ON condition. These results highlight that patients with higher intensity of levodopa-induced dyskinesia, particularly in the axial region, had a poorer voice quality.

In the ON condition, a positive correlation between the total CDRS score and the shimmer (*p* = 0.01, *r*-value: 0.36; 95% CI 0.08–0.60) was found in 44 patients (acoustic data related to ON-medication shimmer were not available for six patients) (Figure 1A). Moreover, the CDRS subscore related to axial (face, neck, and trunk) dyskinesia strongly correlated with the NHR in the ON condition (*p* = 0.01, *r*-value: 0.35; 95% CI to 0.06–0.59) and the shimmer (*p* < 0.01, *r*-value: 0.43; 95% CI 0.16–0.65) (Figures 1B,C). These results highlight that patients with higher values of LID, particularly in the axial region, had a poorer voice quality, objectifying somehow the impact of LID on orofacial activity and the pneumo-phono-articulatory system.

## Discussion

The main aim of this study was to assess the acute effects of levodopa on speech parameters and their possible correlation with PD motor features, LID, and speech parameters in patients with advanced PD. In the acoustic parameters, we found an improvement of the MPT only in female patients, whereas all other parameters were not influenced by medication. This is in trend with previous observations where it was demonstrated a little or no effect of levodopa on speech acoustic parameters (4, 5, 8, 13), maybe in relation to an involvement of non-dopaminergic circuits in the pathophysiology of dysarthria in PD (5). The short-term improvement of the MPT by the levodopa in PD patients was demonstrated previously, supporting the hypothesis that levodopa might improve the thoracic mobility in PD patients (24). However, possible effects of gender on MPT changes after levodopa intake were not investigated (24). Considering our results, it is interesting to note that even if MPT was found to be reduced in both males and females PD patients compared to healthy controls (22), the response of MPT to levodopa appears to be gender dependent. This gender dependency has been previously reported also for other voice parameters such as articulatory acceleration, underlaying the need of gender-based comparison in the analysis of levodopa effect on speech variables in PD patients (25). However, future studies will be needed to confirm these findings. Furthermore, we cannot exclude that the improvement in MPT of sustained phonation in female patients might be also related to a gender-dependent difference in the dose of levodopa per kilogram, regardless that it was not associated with an increase in LID. This hypothesis should be tested in future studies, investigating the possible role of the levodopa dose per kilogram body weight on speech in PD.

Exploring the relationship between the UPDRS scores and speech parameters, we found a negative correlation between the speech item of the UPDRS part III and the MPT in the OFF condition, a measure of the aerodynamic efficiency of the vocal production related to a laryngeal dysfunction or decreased respiratory volume (24). Based on this correlation, we can suppose that aerodynamic efficiency reduction of the vocal tract with the subsequent development of short phrases and short rushes of speech could influence the physician clinical rating of severity of hypokinetic dysarthria, probably even more than reduced loudness and hypophonia. Conversely, as also reported by other authors (26), the speech item in the ON condition correlated positively with the ON shimmer, meaning that patients with greater speech involvement showed poorer voice quality after levodopa intake. Based on these results, we might speculate that voice quality could be one of the main factors that influence the physician clinical rating of speech in the ON state.

Furthermore, we found a positive correlation between the UPDRS axial subscore and the mean frequency of spontaneous speech and sustained phonation in the ON state, meaning that patients with greater axial involvement have a higher mean frequency of voice. In the literature, it has been already showed that hypokinetic dysarthria strongly correlates with axial symptoms (5). However, other authors, evaluating the correlation between the mean F0 of sustained phonation and parkinsonian signs, did not find any correlation with axial scores (27, 28). The considerable inter-subject variability and gender difference of mean frequency might explain those discrepancies. Evaluating the possible correlation between voice parameters and LID, we found a strong correlation between the CDRS axial subscore of LID and shimmer. This means that, in our cohort, patients with higher severity of LID, particularly in the axial region, had a poor voice quality. This has been already reported by other groups that assumed that levodopa could induce an exacerbation of dysarthria in the ON condition by the generation of dyskinesia that can have deleterious effects on speech (29). In this setting, some authors investigated the sequential changes of respiratory, articulatory, and phonatory speech characteristics across a levodopa drug cycle, finding that vital capacity and shimmer had an irregular pattern during the levodopa drug cycle especially at the time of peak motor performance (30). They assumed that the occurrence of dyskinesia could be accompanied by decrease of vital capacity and vocal stability (shimmer), which probably explained the irregular time curves in those parameters during peak motor performance (30). Bearing in mind the abovementioned information and the correlation between the ON axial CDRS score and acoustic indices of speech quality (i.e., shimmer local dB and NHR) found in our cohort, we may suppose that dyskinesia could negatively influence the pneumo-phono-articulatory system (i.e., orofacial, diaphragmatic, chest wall, and abdominal muscles) leading to an abnormal increasing of variation in vocal intensity and a subsequence worsening of speech quality after levodopa intake. Despite data available in large cohorts of patients (2, 8–10), nothing has been reported so far regarding the relationship between LID and acoustics speech parameters through a quantitative analysis, as in our study. Our data underlines that the mixed and heterogeneous effects of levodopa on acoustic indices of speech quality depend on multiple factors (i.e., PD clinical phenotype, characteristics of hypokinetic dysarthria in OFF, and response to levodopa) including LID. We found also an important role of axial dyskinesia that might be cause a dysregulation on the pneumo-phono-articulatory system. The correlation between LID and acoustic speech parameters underlay the necessity of looking for orofacial and axial dyskinesias following levodopa intake, which would induce impact in functional communication and a subsequent significant negative impact on quality of life. Moreover, our results are important to support the need of an accurate clinical assessment of speech disturbances and LID in PD patients who complained speech worsening after levodopa intake. Indeed, in this subgroup of patients, it might be useful to change the patient's treatment by reducing or redistributing levodopa daily doses or introducing anti-dyskinetic drugs such as amantadine. Moreover, in the setting of preoperative evaluation for STN-DBS, it could be particularly important to assess the possible influence of LID on speech parameters in the ON condition. Indeed, patients with a preoperative worsening of speech in the ON medication mainly due to LID may benefit from surgery due to the postoperative LID reduction. On the contrary, patients with a poor ON-medication speech quality before surgery, mainly related to the unresponsiveness of axial PD symptoms to levodopa and not to LID, might not benefit from surgery. Our study has some limitations, mainly related to the retrospective origin of data and lack of a control group to be compared with PD cohort to evaluate the pathological nature of speech parameters between PD patients and healthy controls. Furthermore, we assessed only advanced PD patients, so further studies are needed to test the relationship between levodopa-induced dyskinesia and speech quality also in early PD patients. In conclusion, our results emphasize that levodopa has poor effect on speech acoustic parameters. Moreover, the intensity and location of LID could negatively influence the ON-state speech quality in PD. These findings may partially explain the heterogeneity and the variable effects of levodopa on hypokinetic parkinsonian dysarthria.

## Data Availability Statement

The data that support the findings of this study are available from the corresponding author, upon reasonable request.

## Ethics Statement

The studies involving human participants were reviewed and approved by the local ethics committee (Comitato Etico dell'Area Vasta Emilia Nord; Protocol number: 0031287/18). The patients/participants provided their written informed consent to participate in this study.

## Author Contributions

FC, CB, AG, and FA were responsible for writing the manuscript. FC, CB, AG, SC, VF, EMe, SP, EMo, FV, and FA were responsible for its drafting. CB, SP, EMo, FV, and FA were responsible for its revision. All authors contributed to the article and approved the submitted version.

## Conflict of Interest

FC received personal fees from Zambon outside the submitted work. EMo has received honoraria from Abbott, Medtronic, and Newronika for consulting and lecturing; she has received an educational grant from Boston Scientific. The remaining authors declare that the research was conducted in the absence of any commercial or financial relationships that could be construed as a potential conflict of interest.
